# Transfusion in the Treatment of Acute Hemorrhages

**Published:** 1894-12

**Authors:** T. J. Crofford

**Affiliations:** Memphis, Tenn.


### TRANSFUSION IN THE TREATMENT OF ACUTE HEMORRHAGES.

BY T. J. CROFFORD, M. D., MEMPHIS, TENN.

Abstract of a paper read by Dr. T. J. Crofford, of Memphis, Tenn., before the Southern Surgical and Gynecological Association, in Charleston, S. C., on November 14, 1894, upon the advantages of the sub-cutaneous injections of fluids over the more complicated process of intra-vascular transfusions in the ordinary cases of acute hemorrhage. His method is described as follows:

"Make a saline solution of two, four or six per cent. of common salt, in an ordinary water pitcher; pour this into an ordinary fountain syringe, into the end of the tube of which an aspirator needle has been inserted. While the water is running through this needle, thrust it through the skin into the cellular tissue of the flank or back of the patient. It will be observed that several ounces or a pint of fluid will have been deposited under the skin in quite a short while. The position of the needle can now be shifted to another location without fully withdrawing it, and another quantity of fluid deposited. This can be done several times, till a quart or more of the fluid has been injected without withdrawing the point of the needle from the original puncture made in the skin.

"It will be argued that the intra-vascular injection is directly into the circulation, and is, consequently, on account of its immediate effect, the better applicable to the crises of the acute hemorrhages which need transfusion most. This may be the case where the necessary apparatus and an experienced operator is at hand and ready to do the operation of transfusion. In other words, where you are looking for a hemorrhage, and have prepared for it beforehand, it may be best in certain cases, but this has not been the case in any of the cases of hemorrhage with which it has been my misfortune to deal.

"When we consider the simplicity of the method which I have above described, that no instruments beyond a fountain syringe and an aspirator needle are required, no operation beyond passing this needle through the skin into the cellular tissue; then when we consider the rapidity with which the fluid is absorbed after hemorrhage, it is very evident that this method is the most feasible, and therefore the most applicable, in by far the greater number of cases encountered, whether surgical, obstetrical or medical varieties of hemorrhage be met with."

He next reported a series of cases, then he called attention to the fact that a considerable number of cases dying from hemorrhage rally somewhat, and then die hours, or even a day or two afterward, from cardiac exhaustion, incident to the lessened blood pressure. He urges the necessity of transfusion in all cases where a considerable quantity of blood has been lost, especially if the stomach is not in condition to retain or absorb liquids.

He then observes that when death takes place on account of the sudden withdrawal of a large quantity of blood from the circulation, it is because the heart fails to act.

The heart fails to act because there is a sudden fall in the blood pressure, which is its continuous direct stimulus.

The diminished blood pressure is relieved within certain limits by the vaso-motor system of nerves stimulating the blood vessels to contract, thereby adapting the vascular apparatus to the lessened volume of blood.

If the hemorrhage is sufficiently slow for the vaso-motor contraction of the blood vessels to keep pace with it, then life can be sustained with a proportionately small quantity of blood.

He next calls attention to the independence between the blood, the vascular apparatus and the nervous system, and observes the indications for treatment are—first, to stop the hemorrhage; second, increase the volume of the circulating medium; and third, to sustain the heart and nervous system during the crisis. He also observes that it will be remembered that the middle coat of the blood vessels contains both non-striated muscular tissue and yellow elastic tissue, the former predominating in the small and the latter in the large vessels. In algid congestions, there is a recession of the blood into the large vessels in the interior of the body, leaving a diminished quantity in circulation. It is possible for one to die with all the symptoms of hemorrhage, the blood being poured into the large vessels, instead of upon the ground. The remedy for present relief of the two conditions would, theoretically, be the same.

